# Enhancing Psychological Interventions for Post-Traumatic Stress Disorder (PTSD) Treatment with Memory Influencing Drugs

**DOI:** 10.2174/1570159X21666221207162750

**Published:** 2023-03-08

**Authors:** Enrico Marchetta, Giulia F. Mancini, Maria Morena, Patrizia Campolongo

**Affiliations:** 1 Department of Physiology and Pharmacology, University of Rome, Sapienza, Rome, Italy;; 2 Neuropsychopharmacology Laboratory, European Center for Brain Research, Santa Lucia Foundation, Rome, Italy;; 3 Department of Medicine, Division of Endocrinology, Leiden University Medical Center, Leiden, The Netherlands

**Keywords:** Extinction, cognitive enhancers, MDMA, fear, psychotherapy, trauma, cannabis

## Abstract

Post-traumatic stress disorder (PTSD) is a chronic psychiatric disease resulting from the experience or witnessing of traumatic events. Persistent PTSD symptoms impair patients’ daily quality of life, jeopardizing sleep, mood, sociability, and arousal. Recommended psychological or pharmacological interventions are effective only in a small portion of patients and often lead to relapse. Thus, there is a critical need to address a lack of advancement in the treatment of PTSD. The combination of psychological interventions, aimed at facilitating the extinction of the traumatic memory, and pharmacological medications, represents a promising tool for PTSD treatment. Timely use of psychotherapy in conjunction with pharmacological treatments, rather than monotherapy, could thus determine a synergistic effect by potentiating the effects of psychological interventions. In such a scenario, drugs that modulate cognitive processes involved in the development and/or persistence of post-traumatic symptomatology could be of great help to improve the outcome of psychotherapies and patients' prognosis. The purpose of the present article is to review the current data available from clinical trials on combined pharmacological treatments with psychological interventions in PTSD therapy. An overview of findings from animal studies that prompted clinical research is also discussed.

## 1 INTRODUCTION

Post-traumatic stress disorder (PTSD) is a chronic psychiatric disease that may develop after experiencing or witnessing a traumatic or life-threatening event [1]. PTSD is characterized by several distressing and fearful symptoms which are associated with a maladaptive response to the traumatic event, and which strongly debilitate and compromise patients’ daily life [2]. Avoidance of trauma reminders, hyperarousal, social impairments, sleep disturbances and alterations in emotion, mood and cognition are the most common hallmark features observed in PTSD patients [3, 4]. The majority of people that experience a traumatic event show at least one of the PTSD symptoms soon after trauma exposure, but they spontaneously recover after a first acute response within a few weeks. However, there is a portion of people in which these symptoms become chronic, leading to the development of PTSD [5, 6]. Differently to other well-established chronic psychiatric disorders (*e.g*., schizophrenia, major depressive disorder), PTSD was not officially introduced in the Diagnostic and Statistical Manual of Mental Disorders (DSM) as a standardized mental disease until 1980 [7], and the characterization and classification of this pathology and its symptoms along with traumatic events have undergone several changes over time [8]. The first two editions of the DSM only described a not well-defined psychiatric syndrome related to severe stressors, still far from the current definition of PTSD. In DSM III and DSM IV PTSD was classified as an anxiety disorder. It was only in the DSM V, the latest edition, that PTSD was separated from the anxiety disorders and moved to a newcomer trauma and stressor-related disorders section [9]. In the last decade, much attention has been given to the cognitive features of this disorder, particularly those related to memory alterations [10]. Evidence from experimental studies has reported altered memory consolidation in the onset, and aberrant memory recall and extinction in sustaining and reinstating of this disorder over time [11-13], thus, providing additional strategies for interventions to improve the current therapies for treating PTSD. Prolonged exposure (PE) and cognitive behavioral therapy (CBT) have been developed as psychological interventions to treat PTSD aimed at promoting memory extinction and reducing reconsolidation of the original traumatic memory [14-16]. Briefly, these approaches help patients to associate the trauma-evoking stimuli with safety, thus facilitating traumatic memory extinction. Unfortunately, these first-line interventions are effective only in approximately 50% of the treated patients, and drop-out and long-term relapse have been frequently reported [17]. Further, PE and CBT demand a long period of time and intensive assistance as one single patient needs to be treated for continued and repeated sessions. The latest research attempts have been focused on the combination of psychotherapy with pharmacotherapy to enhance PE and CBT efficacy, especially in the long term to prevent relapse [18-21]. Particularly, enhancing extinction or blocking reconsolidation by combining psychological and pharmacological interventions has been reported as a very promising approach for improving PTSD treatment [12, 13, 22]. Besides traditional drugs acting on neural mechanisms of memory extinction or reconsolidation, there are other compounds known as psychotherapeutic process catalysts that have shown promising results. In fact, such drugs have psychedelic effects, give a sense of well-being, increase extroversion, openness to new experiences, sociability, self-confidence, and attachment behavior, and maintain an optimal arousal state through which PTSD patients may deal with fear memory evoked during psychotherapy without being overwhelmed by anxiety while revisiting traumatic experiences [23].

In this review, we will first briefly describe the three fear memory phases (*e.g*., consolidation, reconsolidation and extinction) that are most significantly altered in PTSD patients, and then we will review data from both human and animal studies on the effects of repeated exposure to trauma reminders on fear memory retention and extinction combined with pharmacological intervention.

## 2 CONSOLIDATION OF FEAR MEMORY

No memory persists for most experiences lived in our own past life. However, for some of them, particularly those related to emotionally, traumatic or life-threatening events, our brain creates memories that may endure from hours or days to even our entire lifespan [24]. This process by means of which memory could be stored and becomes stable over time is known as memory consolidation [25]. This is characterized by two sequential and distinguished processes. The first, “synaptic consolidation”, is accomplished within a few minutes to hours after the encoding of information and, as the name suggests, it is characterized by functional and structural changes at the synaptic level (*e.g*., cross-talk between synapses, cell body and nucleus) [26]. New gene expression and protein synthesis are thought to modulate memory consolidation [27, 28]. In particular, cyclic adenosine monophosphate (cAMP) responsive element binding protein (CREB), after being activated by upstream transduction factors, such as protein kinase A and Ca^2+^/calmodulin-dependent protein kinase IV, induces the expression of CRE-regulated genes, including a number of immediate-early genes, such as transcription factors, that in turn regulate the expression of late response genes [25, 29, 30], giving rise to several neuronal changes culminating in synaptic remodeling, which is assumed to convert short-term memory to a long-term one. Emotionally arousing experiences are generally well remembered [31]. Epinephrine and cortisol, released by emotional arousal, strengthen the persistence of memory of significant experiences [32]. Epinephrine does not cross the blood-brain barrier (BBB) and modulates memory consolidation by activating β-adrenergic receptors on peripheral vagal afferents projecting to the nucleus of the solitary tract in the brainstem. Glucocorticoids (GCs) released from the adrenal cortex rapidly enter the brain by crossing the BBB and then activate glucocorticoid receptors (GR) [33]. The mechanism of synaptic modulation of memory consists of multiple non-genomic and genomic processes, resulting in increased synaptic strength of neurons in the basolateral amygdala (BLA) [24]. These effects are caused by a rapid release of presynaptic glutamate and enhanced expression of α-amino-3-hydroxy-5-methyl-4-isoxazolepropionic acid (AMPA) glutamate receptors in postsynaptic membrane after stimuli. In turn, BLA projections induce synaptic plasticity in other brain areas (*e.g*., striate nucleus, hippocampus) [34]. The neuroendocrine system, notably the hypothalamic-pituitary-adrenal axis (HPA-axis) and the noradrenergic system, have been strongly implicated in the pathophysiology of PTSD [35]. Based on preclinical studies, the monoamine hypothesis of PTSD has been enlarged with a more complex neurochemical and neuroplasticity hypothesis that highlights the role of the glutamatergic system in trauma and stress psychopathology [36]. The second stage is the “systems consolidation” which, if compared to “synaptic consolidation”, is considerably slower. It takes weeks, months, or even years to be accomplished and differently to synaptic consolidation it appears to be not hippocampal-dependent, although literature data have shown an involvement of this brain area as well [37-39]. Systems consolidation is the process through which memories from the hippocampus, where they are initially encoded and stored, are reorganized, and relocated to the neocortex, thus allowing memories to persist permanently [38]. The mechanisms sustaining systems consolidation have not been fully elucidated. It seems that over time, the recurrent activation of the memory trace within the hippocampus either in explicit recall or in implicit processing (*e.g*., during sleep), induces the activation of projections from the hippocampal formation and related structures to neocortical neurons, triggering the synaptic consolidation locally [38].

## 3 RECONSOLIDATION OF FEAR MEMORY

The consolidation theory of memory assumes that the process by which an experience as a labile trace becomes a stable memory can occur just once [40]. This means that the events at the synaptic and cellular level, which allow an experience to be initially encoded and then stored in the long term, are immutable. However, some studies questioned the validity of this assumption [41, 42], by asserting that the retrieval of a consolidated memory (*i.e*., remembering the events that were previously stored in the brain) can restore its labile form. In particular, it has been demonstrated that after retrieval, a memory trace is susceptible to interference, which may disrupt the original consolidated memory [43, 44]. Therefore, an additional consolidation phase is necessary to stabilize the memory trace once again [45]. This mechanism is known as the reconsolidation phase of memory, and differently to what the name might suggest, it is not a simple replication of the consolidation phase, although overlaps between the underlying mechanisms of these two phases exist [46]. Studies in rodents have demonstrated that memory may be disrupted *via* inhibition of new gene expression or protein synthesis in the hippocampus immediately after retrieval of a consolidated memory [47, 48], whereas the same manipulations into the hippocampus long after retrieval had no effect on memory [49]. This indicates that these manipulations are effective only when given soon after reactivation but not when given after a delay. In addition, consolidated memories that are not retrieved, and therefore do not become labile, cannot be disrupted by the inhibition of gene expression or protein synthesis [50]. Hence, if a consolidated memory could turn back to its labile state and be disrupted by external interventions, some authors argue that there might be the chance that the disruption of the original traumatic memories could be an efficacious approach to treat PTSD patients [51, 52].

## 4 EXTINCTION OF FEAR MEMORY

The extinction of fear memory is the process through which the fear response, resulting from the retrieval of traumatic memory, is mitigated by an inhibitory learning mechanism [53]. Extinction is, hence, not the same as unlearning. Memory extinction was first pointed out by Pavlov in 1927 and it has been largely investigated in rodents [54]. In particular, the temporal association of a neutral stimulus (conditioned stimulus, CS; *e.g*., tone, light or context) with an aversive one (unconditioned stimulus, US; *e.g*., footshock) determines a conditioned fear response (*i.e*. freezing behavior) when the CS is subsequently presented alone. However, repeated CS presentations without footshocks (extinction training) were shown to gradually dampen the conditioned fear response involving extinction memory process [55]. Extinction training is not the same as forgetting, but it is considered as new learning because it leaves the CS as having two meanings: “fear” if coupled with the US or “safe” if it is not coupled with US. When memory is extinguished, it means that the “safe” prevails over the “fear” meaning [56]. Many studies have provided evidence demonstrating that extinction consists of a new learning process. Specifically, without extinction training, the fear response lasts months or even years [57], and extinguished fear responses return naturally with the passage of time (spontaneous recovery) [54, 58], after using a different context compared with the one in which extinction training took place (renewal) [59], or as a consequence of the re-exposure to the US (reinstatement) [60-62]. In clinical practice, extinction learning is engaged during PE sessions. Similarly to what has been documented in animal models of conditioning and extinction, PE aims at dampening the fear response through repeated presentations of harmless but feared stimuli without the presence of adverse consequences [63]. During exposure therapy, feared stimuli can be presented in several ways: through the patient’s imagination, in real life, or in virtual reality [64]. This process is thought to create a new memory trace that prevents the re-emerging of the original fear memory when cues related to the adverse consequences are encountered [65]. In this scenario, medications whose mechanism of action determine an enhancement of the extinction learning after exposure therapy could be timely used to potentiate and expedite this psychological intervention, thus reducing drop-outs, relapses and recrudescence of the pathology. In the following sections, we will describe the potential efficacy of combining pharmacological treatment with repeated exposure to fear stimuli on the improvement of fear memory alterations seen in PTSD at both clinical and preclinical levels. Specifically, we will focus on the effects induced by D-cycloserine, yohimbine, methylene blue, glucocorticoids, cannabinoids, hallucinogens and psychedelics.

## 5 MATERIALS AND METHODS

Literature search was conducted in March, 2021 by comprehensive searches in two online databases (PubMed and Scopus). The keywords used for the search of animal studies were: *D-cycloserine, yohimbine, methylene blue, glucocorticoids or corticosterone or methyrapone, cannabinoids or endocannabinoids, MDMA or 3-4, methylenedioxymethamphetamine, ketamine, psychedelics and/in post traumatic stress disorder or posttraumatic stress disorder or post-traumatic stress disorder or fear memory or memory extinction or memory reconsolidation*. Results were limited to rodents studies. The keywords used for the search of human studies were: *D-cycloserine, yohimbine, methylene blue, glucocorticoids or cortisol, cannabinoids or endocannabinoids, MDMA or 3-4, methylenedioxymethamphetamine, ketamine, psychedelics and/in post traumatic stress disorder or posttraumatic stress disorder or post-traumatic stress disorder*. For both the 1^st^ (*i.e*., titles and abstracts) and the 2^nd^ (*i.e*., full-text articles) screening phases, the following exclusion criteria were used: (1) papers written in a language other than English; (2) non-original researches (*e.g*., reviews, commentaries, editorials, book chapters); (3) no full-text articles (*e.g*., meeting abstracts); (4) studies *in vitro*; (5) studies in non-human animals other than rodents; (6) drugs administration not associated with an animal model of PTSD or psychological intervention in humans; (7) absence of control groups in the studies.

## 6 IMPROVING EFFECTS OF PSYCHOTHERAPY COMBINED WITH DRUGS: EVIDENCE FROM ANIMAL AND HUMAN STUDIES

### 6.1 D-cycloserine

#### 6.1.1 Animal Studies

**Fig. (1):**
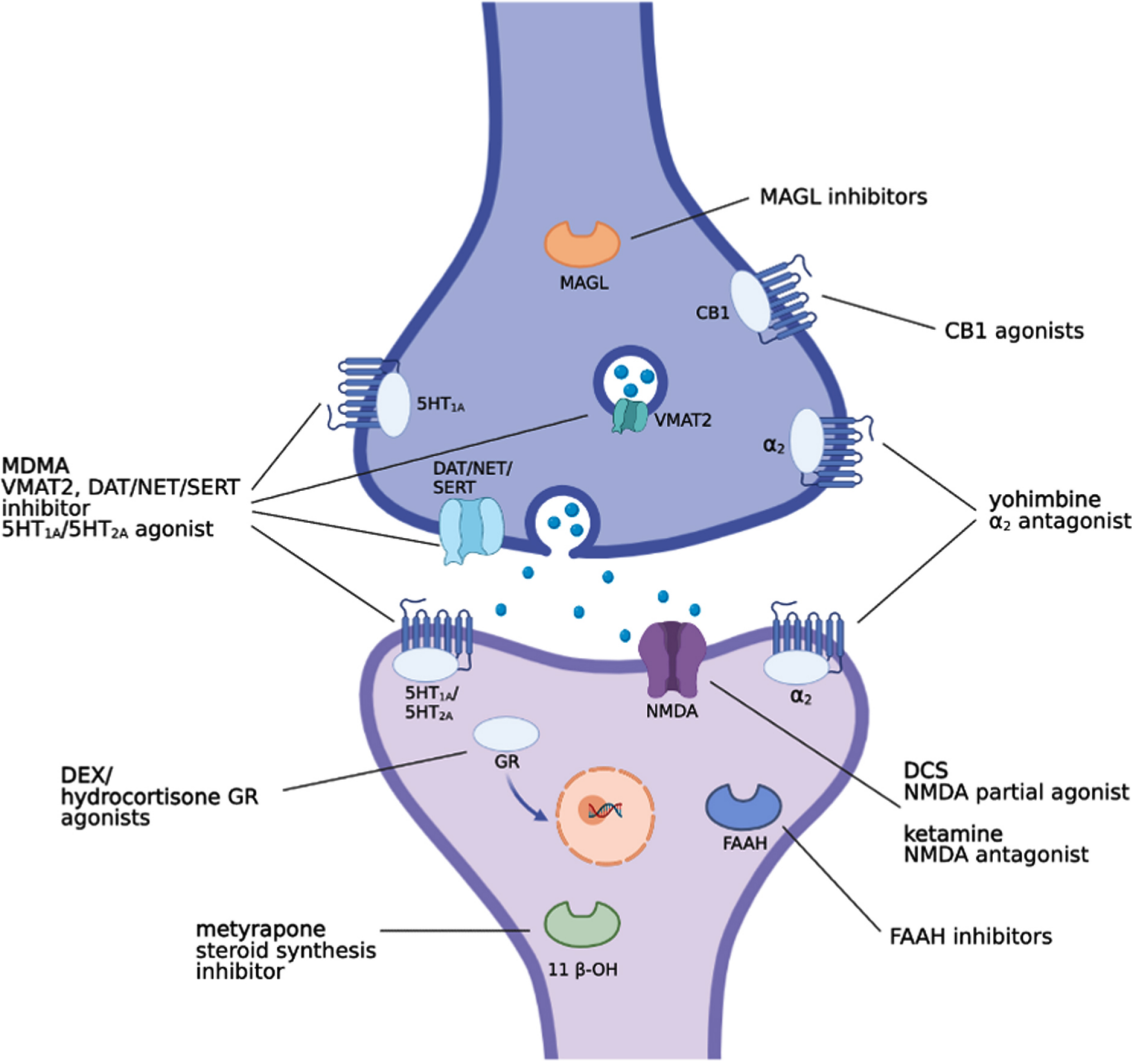
Representative image indicating the main mechanisms of action of DCS, yohimbine, glucocorticoids (DEX and hydrocortisone), cannabinoids and hallucinogens (MDMA and ketamine).

The engagement of D-cycloserine (DCS), a N-methyl-D-aspartate (NMDA) receptor partial agonist (Fig. **1**), in association with exposure therapy for treating PTSD and other mental disorders in humans comes from preclinical evidence. Walker *et al.* (2002) trained rats to associate a light (CS) with a footshock (US), in a cued fear conditioning paradigm. 30 min before sessions of light presentations in the absence of the US (*i.e*., extinction session), animals were intraperitoneally (i.p.) administered with DCS (3.25, 15 or 30 mg/kg), which dose-dependently facilitated extinction of conditioned fear. It was found that DCS acted at the strychnine-insensitive glycine-recognition site of the NMDA receptor complex and was effective only in rats that received combined extinction training [66]. Starting from the evidence that NMDA antagonists reduced fear extinction retention [67, 68], it was hypothesized that DCS could reduce fear memory expression. It was found that DCS administration improved extinction retention only when administered after the extinction training, suggesting that NMDA receptors are implicated in the consolidation and not in the retrieval of extinction memory [68]. In addition, increasing the delay between the end of the extinction training and DCS administration linearly reduced the enhancing effect of DCS on extinction learning [69]. This evidence identifies the existence of a crucial and optimal time window for certain drug administration when combined with psychological interventions. Another study demonstrated that fear memory extinction in drug-free condition is normally selective for one specific cue, while the effect of DCS seemed to generalize across different conditioned stimuli [70]. Since PTSD is typically associated with multiple fear cues, the generalization effect of DCS used in conjunction with exposure-based psychotherapy could be very beneficial and largely exploitable in clinical practice. Another study also demonstrated that DCS facilitated extinction retention of an aversive odor-cue in a time-dependent manner [71], further confirming the cruciality of the timing for DCS administration, which has been shown to be effective only when given in a limited time window post-extinction training. This demonstrates that DCS facilitates extinction only if the behavioral procedure first engages the extinction learning process. These findings were additionally supported by Bouton and colleagues [72] by showing that only rats that engaged extinction learning process, that is, rats with higher extinction learning, benefited from the improving effect of DCS, whereas rats with lower indices of extinction learning did not. When animals presented poor extinction learning, DCS was found to have no improving [72] or even detrimental effects [73]. Taken together, the fact that DCS has been reported to be effective only when given after extinction learning (*i.e*., after psychotherapy sessions, translated to humans) represents a potential advantage for DCS therapy since it would not require daily treatment, thus improving the compliance, and prevent the occurrence of tolerance which has been reported after repeated administrations [74].

#### 6.1.2 Human Studies

The first study showing the efficacy of DCS in improving the effectiveness of exposure therapy was conducted by Ressler and colleagues in patients with height phobia [75]. Exposure therapy combined with DCS resulted in a significant reduction of acrophobia symptoms and posttreatment skin conductance fluctuations during a virtual reality exposure to heights compared to placebo. Further, other studies have demonstrated that DCS improved the efficacy of exposure therapy also for other mental disorders, such as social anxiety disorder [76, 77], panic disorder [78], and obsessive compulsive disorder [79]. PE therapy, however, requires multiple sessions leading to high drop-out rates, thus, in accordance with preclinical findings, a combination of PE with DCS could accelerate the remission of PTSD and shorten the overall duration of PE therapy [80]. In addition, due to its limited cost and slighter side effects compared to the currently used drugs in PTSD treatment (*e.g*., benzodiazepine and SSRI, which are associated with low compliance and high drop-out rate [81]), DCS may be a valuable clinical option. Importantly, DCS or other medications that facilitate memory extinction could switch the current symptomatic intervention, whose efficacy rate is typically low, in an etiological and resolutive therapy [82]. The synergistic improving effect of DCS on the efficacy of exposure therapy in PTSD patients has been tested in a double-blind randomized controlled clinical trial [83]. Twenty-six veterans of the Iraq and Afghanistan wars diagnosed with PTSD were randomly assigned to exposure therapy plus DCS or placebo. Participants attended a total of 6 sessions of 60-90 min each. The DCS group received 50 mg of DCS 30 min prior to sessions 2-5 that entailed imagine exposure, whereas the placebo group received a placebo pill on these four occasions. At assessment, patients in the DCS group reported significantly worse PTSD and depression outcomes compared to the placebo group. In addition, it was found that compared to the placebo group, DCS-treated patients had an increase in self-reported distress beginning with the second exposure session, suggesting that DCS might increase fear learning when administered before exposure sessions [83]. This clinical report corroborates preclinical findings highlighting the considerable importance of the time window of drug administration again when combined with psychological interventions. Thus, DCS could enhance the extinction or reconsolidation of traumatic memories depending on when its administration occurs. Indeed, when a memory is reactivated, it becomes temporarily destabilized and alterable. A high fear state could lead to the reconsolidation of fear memory in a stronger state, worsening, instead of improving, PTSD symptoms. Therefore, DCS should be administered in a timely manner taking into consideration the time frame of memory mechanisms (reconsolidation or extinction) and the pharmacokinetic properties [83]. Conversely, other studies carried out in adults [84, 85], and in pediatric patients [86] did not find any additional benefit of DCS treatment combined with exposure therapy on primary outcomes. Rothbaum and colleagues, by using virtual reality/prolonged exposure (VR/PE) sessions, found that PTSD symptoms significantly improved from pre- to post-treatment until 3, 6, and 12 months of follow-up [85], but they did not find any ameliorative effects in DCS-treated patients compared with those treated with placebo, over VR/PE sessions on primary outcomes. However, in secondary analyses between baseline and six-month follow-up, they revealed that DCS treated patients reported lower startle response and salivary cortisol concentrations during VR scenes than placebo treated patients. de Kleine and colleagues found that PE effectively reduced PTSD symptoms without an overall enhancement effect for combination with DCS (50 mg/d) [84]. However, a subgroup of patients who responded more slowly to PE sessions, reported a greater symptom reduction over sessions if treated in combination with DCS [84]. Conversely, DCS improved the efficacy of VRE in reducing PTSD symptoms as well as on secondary outcome measures of sleep, depression, and anger expression compared with the VRE placebo group by the end of treatment [87]. It is important to underline that, differently to the above unsuccessful studies, after adjustment of the experimental protocol, more successful results were obtained. For instance, DCS was administered in a higher dosage (100 mg/d) and longer before (90 min) the start of the therapy, compared to the above studies in which DCS was dosed at 50 mg/d and given 30-60 min before the beginning of the therapy. In addition, the authors enrolled 25 civilians with trauma related to the 9/11 World Trade Center terrorist attack and used a longer experimental protocol that engaged 12 VRE sessions compared to the 6 sessions used in the above-described unsuccessful studies. Interestingly, these results are in line with a recent metanalysis in which the association between DCS administration parameters and treatment outcomes were investigated in patients with anxiety disorders [88]. Several studies have observed that DCS efficacy was dependent on the level of fear that patients reported at the end of the session, by showing that DCS may enhance extinction learning when fear is low, but it may increase fear learning *via* reconsolidation if fear remains high by the end of an exposure session [89, 90]. However, these findings were not confirmed by Smits and colleagues [91] that evaluated the efficacy of DCS in enhancing exposure therapy outcomes when administered to patients with social anxiety disorders whose fear was low by the ending of the session (tailored DCS administration strategy). More studies are needed to confirm these results and future research attempts should take into account that unlike anxiety or phobias, PTSD is typically associated with multiple fear cues, negative mood and cognition deficits, as well as hyperarousal and insomnia, all of which may contribute to impair extinction learning [1]. Further, due to the potential ceiling effect arising from the high efficacy rate of psychotherapy, the number of enrolled participants must be large enough to detect statically significant results. For instance, selecting patients whose PTSD symptoms resulted resistant to psychotherapy may unveil the advantages of using DCS coupled with exposure therapy, avoiding ceiling effects.

### 6.2 Yohimbine

#### 6.2.1 Animal Studies

The consolidation of newly acquired information and the extinction of a consolidated memory share many common mechanisms. A large body of literature has reported that the noradrenergic system is involved in both memory consolidation and extinction [24, 92-94]. Early studies reported an impairment in fear extinction after neurochemical lesion of locus coeruleus, a small pontine nucleus that presents a high concentration of adrenergic cell bodies [95, 96]. Further, the infusion of the β-adrenergic receptor antagonist propranolol into the infralimbic region of the rat prefrontal cortex (PFC) prior to extinction training impaired later retrieval of the extinction memory [97]. Yohimbine, an antagonist of both pre- and post-synaptic adrenergic α2-receptors (Fig. **1**), facilitates fear extinction [98, 99]. However, it impaired the extinction of a cocaine-conditioned place preference (CPP) task in mice with a mechanism that seemed to be independent of adrenergic α2-receptors [100]. Indeed yohimbine has been reported to also activate dopaminergic D2 and multiple serotonin (5-HT) receptor subtypes displaying a complex mechanism of action [101-105]. Thus, these results suggest that the extinction of a CPP task is not dependent on adrenergic α2-receptors, but it could be dependent on other different receptors. It is possible that the mechanisms underlining the extinction of a CPP task are completely different from those of the extinction of fear memory, and thus it could explain why the extinction process of a CPP task is not dependent on adrenergic α2-receptors. The first study examining the role of NE neurotransmission in the extinction of conditional fear was reported by Cain and colleagues [106]. They found that yohimbine (5 mg/kg, subcutaneously; s.c.) administered prior to, but not immediately after, extinction training facilitates long-term cue and contextual fear extinction retrieval. The extinction-facilitating effect of yohimbine also occurred in mice that underwent temporally spaced extinction training (7 CSs presentations separated by 20 min of inter-trial), a procedure that was found to impair long-term fear extinction [106]. These effects seem to be not sex- or species-dependent since female rats trained to pair a tone with a footshock and injected with yohimbine (1 mg/kg, i.p.) prior to extinction training showed improved extinction learning [107]. Similarly, Mueller and colleagues [108] showed that yohimbine reduced freezing in male rats during extinction training, but it did not facilitate fear extinction retrieval when rats were tested the next day. It is well-known that the extinction of a cue-conditional fear also depends on the context in which extinction training is performed. In other words, if the subject received the conditioning in context A and then the extinction training in context B, the fear might be “renewed” when extinction retention is tested in either context A or even in a novel context C [59, 109, 110]. Interestingly, Morris and Bouton also demonstrated that yohimbine (1 mg/kg, i.p.), administered prior to extinction learning in female rats, fails to prevent renewal of fear memory when extinction retrieval was tested in a different context than the one in which extinction learning had originally occurred (*i.e*., the original conditioning or a novel context) [107].

#### 6.2.2 Human Studies

Evidence has demonstrated that the higher the emotional activation during exposure sessions, the more positive outcomes of the therapy and *vice versa*. [111-113]. Yohimbine enhances the release of NE from locus coeruleus, increasing NE levels within brain areas crucially associated with fear conditioning and extinction, such as the amygdala, hippocampus, and PFC [114, 115]. While the efficacy of some cognitive enhancers relies on direct modulation of extinction learning (*e.g*., DCS), yohimbine was proven to act by enhancing emotional engagement during the exposure sessions [116]. Yohimbine improvement of exposure therapy efficacy has been tested in two studies that enrolled patients with claustrophobic fears and social anxiety disorder [115, 117]. To date, only one study has reported the effect of yohimbine combined with exposure therapy for PTSD patients [118]. Twenty-six male combat veterans aged 18-45 underwent a randomized placebo-controlled double-blind clinical trial. Oral yohimbine HCl (21.6 mg) was dosed one hour prior to the first imaginal exposure (IE) of a PE protocol. Yohimbine administration resulted in a high emotional engagement with increased heart rate, blood pressure, physiological arousal, and subjective distress during the first image exposure session compared to placebo. One week after administration, the authors found a reduced trauma-cued heart rate reactivity, a steeper slope of improvement for Beck Depression Inventory-II over the course of treatment, and greater between- and within-session declines in distress, though no benefits on PTSD severity at the end of a standard course of PE was observed. Exploratory outcomes suggested that the amount of yohimbine-related arousal was positively correlated with subsequent clinical responses. It was thus hypothesized that to have an effective extinction learning, the corrective information during the exposure session must be paired with an enhanced emotional engagement. Although the sample size was relatively small, and these results should be confirmed in a larger sample, the authors conclude that yohimbine, by increasing the emotional engagement within the exposure sessions, improved the efficiency of PE therapy. Many concerns about the association of oral yohimbine with panic attacks and flashbacks were raised in other studies [119, 120], and this limits the use of yohimbine in clinical practice for PTSD treatment.

### 6.3 Methylene Blue

#### 6.3.1 Animal Studies

Although methylene blue (MB) has been used for many years in therapy, its use as an extinction enhancer is fairly recent. Differently to DCS and other cognitive enhancers, the improving effects of MB on extinction learning consolidation are not due to direct modulation of a neurotransmission system but to enhanced metabolism following increased mitochondrial respiration [121, 122]. This leads to an enhancement of oxygen consumption and an enrichment of adenosine triphosphate availability within cells. MB boosts the metabolism preferably in neurons whose energy demands are high, like those activated during the extinction learning, regardless of which neurotransmission is involved [122]. MB was found to enhance memory retention in spatial memory and object recognition tasks [123, 124]. Closer to our purpose is the evidence according to which MB administered during the 5 days following an extinction training enhances memory retention of extinction of a Pavlovian fear conditioning in rats [125]. Learned helplessness is a phenomenon that develops in animals after being subjected to footshock and inhibits animals from learning an escape response [126]. Just as PTSD patients, rats showing learned helplessness resemble some behavioral features of the human pathology, such as decreased reward sensitivity, increase in a conditioned fear, and alteration of fear extinction [127]. Similarly to what has been observed in humans, where PTSD develops only in 5-10% of individuals who experienced a traumatic event, also animal models show individual variability to develop comparable behavioral outcomes following a traumatic experience [128]. Such stress vulnerability may have a genetic or epigenetic basis that can be inherited to the next generations. Experiments on congenitally helpless rats, selectively bred until the 25^th^ generation when 95% of rats showed a helpless behavior phenotype, presented exaggerated renewal and extinction learning deficits [129]. Daily doses of MB (4 mg/kg; i.p.) enhanced retention of the extinction memory as demonstrated by significant low fear renewal compared to saline-administered congenitally helpless rats [129]. Additionally, a separate study demonstrated that the extinction memory enhancement induced by MB was an effective strategy for reducing fear reinstatement in Long-Evans rats genetically selected for displaying enhanced extinction learning [130].

#### 6.3.2 Human Studies

Similarly to what has been observed in rodents, it appears that in humans with claustrophobic fear MB enhances the retention of fear extinction when administered after a successful exposure session but has a deleterious effect on extinction if participants present high levels of post-session fear [131]. To date, only one study has assessed the effect of MB after exposure therapy sessions in PTSD patients [132]. In this double-blind randomized study, 42 PTSD patients were enrolled and subjected to 6-sessions of 50-min daily IE protocol. The effects of MB (260 mg), dosed after 2-6 exposure sessions, were compared to placebo and a second waitlist control group receiving a full PE protocol after a gap of 1 month. They found that brief IE with MB or placebo yielded no difference across multiple indices compared to the full PE protocol. In addition, it was revealed that there were small to moderate differences favoring IE + MB over IE + placebo at follow-up. It is important to consider that this was a small clinical trial with moderate effect sizes and limited generalizability of its findings. Taken together, findings from preclinical and clinical studies summarized above indicate that, similarly to what has been observed with DCS, only those patients capable of showing some extinction seem to benefit from MB treatment. MB increases the metabolism of activated neurons indiscriminately, so it could promote a stronger extinction memory consolidation but also a reconsolidation process when the patients’ fear is high at the end of the exposure sessions as well, thus, leading to an enhancement, instead of inhibition, of the fear memory recall over time [131]. Nonetheless, since MB can be administered at the end of an exposure session without losing its effectiveness, it remains suitable for treating only those patients whose fear at the end of an exposure session is low and, thus, avoiding any possible fear strengthening effects.

### 6.4 Glucocorticoids

#### 6.4.1 Animal Studies

Evidence from both human and rodent studies has reported that GCs play a key role in learning and memory processes. Both corticosterone in animals and cortisol in humans enhance the consolidation of fear memory [133-136] which can subsequently lead to the development of PTSD [137]. Accordingly, metyrapone, a steroid synthesis inhibitor by targeting the enzyme 11 β-hydroxylase (11 β-OH) (Fig. **1**), impaired memory consolidation in both animals and humans [138, 139]. Overall, consolidation and extinction of fear memories require overlapping molecular pathways and neural circuitry among the amgydala, mPFC and hippocampus [140-143]. Nonetheless, differences that characterize consolidation and extinction of fear memory as two distinct phases exist [144, 145]. Therefore, it was hypothesized that as it happens for the consolidation of a new memory, GCs could enhance the consolidation of fear memory extinction as well [133, 135]. Several studies have demonstrated that GCs administration enhanced the consolidation of fear memory extinction in several types of behavioral paradigms, including auditory fear conditioning [146], contextual fear conditioning [147, 148], fear-potentiated startle [149], and in the predator stress paradigm [150], whereas agents that suppress glucocorticoid signaling impaired it [146, 147, 149, 150]. In particular, mice exposed to tone-footshock pairings were treated with metyrapone (50 mg/kg; s.c.) or saline 90 min before an extinction training which consisted of 60 tones presentations without footshocks. Extinction learning retention was conducted in the extinction context and in the acquisition context, 1 and 3 days after extinction training, respectively [146]. Metyrapone reduced the retention of the fear memory extinction in both the extinction context and in the acquisition context without affecting the memory performance during the extinction training. Similarly, mice trained to fear a novel context with footshocks, were injected 48-h later with various doses of corticosterone (0.3, 1.0, 3.0, and 10.0 mg/kg; i.p.), the protein synthesis inhibitor anisomycin (150 mg/kg; i.p.), or vehicle 5 min after the context-induced reactivation of fear memory. The following day mice were tested for contextual fear memory retention [148]. Only corticosterone at 3.0 mg/kg and 10.0 mg/kg administered after extinction training decreased contextual fear memory 24 h later as significantly as anisomycin did. Corticosterone seemed to reduce the contextual fear memory by acting on extinction rather than reconsolidation process. GCs had indeed no effect on subsequent memory recall neither after an extended time window (72 h) nor when corticosterone was injected in absence of context-induced fear memory reactivation [148]. In addition, as expected, the effect of corticosterone was completely reversed by interposing a reminder shock 4 h after context-induced fear memory reactivation. Moreover, 3 days of repeated corticosterone administration before and after context-induced fear memory reactivation led to decreased fear memory expression the next day in drug-free condition. Thus, this evidence demonstrated in mice the potential clinical implications of using corticosteroids during exposure-based therapies where multiple exposure sessions are normally used [148]. The effects of GCs on extinction of fear memory were also evaluated by using fear-potentiated startle [149]. The GR agonist dexamethasone (DEX; Fig. **1**) administered at different doses (0.1, 0.5, 1.0 mg/kg; i.p.) dose-dependently facilitated extinction of conditioned fear, but DEX at the dose of 1.0 mg/kg produced the maximal enhancing effect in extinguishing fear memory. On the contrary, the administration of metyrapone significantly blocked the extinction of conditioned fear, whereas the concomitant administration of DEX restored the same extinction magnitude as in rats treated with DEX alone [149]. These effects seemed to be mediated by GR in BLA, in which participation of the NMDA receptor was also demonstrated [151]. Additionally, the ameliorating effects of corticosterone on fear memory extinction have been shown to be long-lasting. Mice trained in contextual fear conditioning and treated with corticosterone (10.0 mg/kg) or vehicle soon after each of the 4 daily extinction sessions, demonstrated not only enhanced extinction of fear memory across the extinction sessions but also 1-week after the last extinction trial [147]. Further, the same study demonstrated the specificity of this corticosterone effects on facilitating fear extinction rather than on blocking the fear reconsolidation process, since a reminder shock, which did not cause significant contextual fear conditioning in naive mice, determined the reinstatement of the extinguished fear memory, completely reversing the pro-extinction effects of corticosterone [147]. Conversely, the corticosteroid synthesis inhibitor, metyrapone, dosed before the extinction sessions, enhanced the fear memory expression across the extinction trials. Such fear enhancing effects lasted at least 1 week and were dependent on the reactivation of the fear memory [147].

#### 6.4.2 Human Studies

It has been demonstrated that GCs enhance memory consolidation of emotionally arousing experiences and promote extinction learning in humans [152, 153]. In addition, GCs have been proven to be useful in reducing patients’ distress during exposure sessions [154]. These effects could be suitably exploited during PE therapy, not particularly for civilian patients but rather for combat veterans with PTSD for whom high dropouts, disturbances in attention and cognitive impairments were revealed [155]. The enhancing effect of GCs combined with PE therapy was firstly reported in a case report in which 30 mg of hydrocortisone, a GR agonist (Fig. **1**), or placebo were orally administered 30 min prior to exposure sessions [156]. Both placebo- and hydrocortisone-treated patients experienced a reduction in PTSD symptoms, but greater improvements were found in a patient treated with hydrocortisone. This was pointed out by both a general assessment of the Clinician-Administered PTSD Scale (CAPS, a 54 point reduction compared to a 42 point reduction achieved by placebo) and self-rated PTSD Symptom Scale (PSS-SR, a 20 point reduction, compared to a 7 point reduction achieved by placebo) and by a specific symptom clusters assessment indicating a substantially greater reduction in avoidance symptoms in the hydrocortisone-treated patients. In addition, placebo-treated patients experienced more distress, low adherence to the therapy and hid some details related to the trauma, which led to inappropriately complete IE. This first evidence was confirmed in a double-blind, randomized clinical trial by Yehuda and colleagues, in which veterans treated with 30 mg oral hydrocortisone 20 min before PE sessions reported a greater reduction of PTSD symptoms over time, despite a significant difference in post-treatment CAPS scores was not observed compared to placebo [155]. The demonstration that participants with higher lifetime PTSD severity were most likely to respond positively to hydrocortisone raised the possibility that patients with a more severe disorder may greatly benefit from a pharmacological intervention that addresses GC responsivity. Patients’ responsiveness to hydrocortisone was, indeed, linked to a greater sensitivity to GCs at pre-treatment, which went towards a significant decline in sensitivity following treatment, thus, suggesting that hydrocortisone may enhance the effectiveness of PE through such “normalization” of glucocorticoid sensitivity [155]. They speculated that elevated glucocorticoid sensitivity may be a functional biomarker for patients who may benefit from a combined therapeutic approach in which manipulations of the HPA axis are affected concurrently with psychological interventions [155]. In a second clinical trial, 27 veterans with a diagnosis of PTSD received 0.5 mg of oral DEX or placebo the night before their VRE sessions [157]. Differently to Yehuda and colleagues [155], the drop-out rate was significantly higher and PTSD symptoms worsened in the DEX group (76.9%) compared to the placebo group (28.5%). Contrary to what was expected, DEX did not enhance exposure therapy outcomes and was associated with increased drop-out. This is not surprising considering that DEX, differently to hydrocortisone, does not activate the central nervous system due to its low permeability through the BBB and its inhibitory control on endogenous cortisol release [157].

### 6.5 Cannabinoids

#### 6.5.1 Animal Studies

Cannabis contains several terpeno-phenol molecules known as natural cannabinoids or phytocannabinoids, among which Δ^9^-tetrahydrocannabinol (THC) and cannabidiol (CBD) are the principal and most studied compounds [158, 159]. While most of the psychoactive effects are due to THC through direct activation of cannabinoid receptors, CBD mechanism of action is more complex and still less known. The endocannabinoid (eCB) system is a neuromodulatory system in our body, composed of two main endogenous ligands N-arachidonoyl ethanolamine (anandamide, AEA) and 2-arachidonoyl glycerol (2-AG), which exert their effects by binding to two metabotropic receptors named cannabinoid type 1 (CB1) and type 2 (CB2) receptors [160, 161]. The eCBs AEA and 2-AG are mainly degraded by two enzymes fatty-acid amide hydrolase (FAAH) and monoacylglycerol lipase (MAGL), respectively. Increasing number of animal studies suggests that eCB signaling is critically involved in the extinction phase of fear memory [162]. Several reports have demonstrated that increased AEA signaling, through inhibition of FAAH activity, induced an enhancement of fear memory extinction [163-165]. On the contrary, the administration of CB1 receptor antagonists impaired the extinction and reconsolidation of fear memory [166, 167]. In accordance with these results, CB1 KO mice or mice bearing the FAAH C385A polymorphism, which impairs the activity of FAAH, showed reduced and enhanced fear memory extinction, respectively, thus, supporting the hypothesis that strengthening AEA signaling at CB1 receptors enhances fear extinction [168, 169]. In a PTSD-like rat model, we have recently demonstrated that the FAAH inhibitor URB597 or the direct cannabinoid receptor agonist WIN55,212-2 (Fig. **1**) enhanced fear extinction tested 16 days post-trauma, when administered after three spaced extinction sessions, 7, 10 and 13 days after trauma exposure [170]. Both drugs showed beneficial effects, but only URB597 (0.1 mg/kg, i.p.) induced the best improvements by enhancing extinction consolidation and restoring normal social behavior in traumatized rats through indirect activation of CB1 receptors tested up to 36-37 days post-trauma. Further, another recent study has confirmed these findings in male rodents showing beneficial effects on fear extinction of the FAAH inhibitor URB597 [171]. Contrarily to what has been found and described above in males, increased AEA signaling at CB1 receptors (Fig. **1**), 60 min prior to extinction training in an auditory fear conditioning paradigm, did not alter fear memory extinction in female rats [171]. However, when AEA signaling was increased at the transient potential receptor of vanilloid type-1 channel (TRPV1R, another target of AEA beyond CB1 receptors), it augmented freezing behavior at extinction training and extinction retrieval. Increased 2-AG signaling at CB1 receptors reduced freezing at extinction training in females, without altering fear responses in males [171].

#### 6.5.2 Human Studies

Similarly to what has been observed in rodent studies, several evidence in healthy volunteers indicate that potentiating cannabinoid transmission might reduce stress reactivity, anxiety and indices of fear [168, 172-175]. Rigorous clinical studies regarding the efficacy of cannabinoids for PTSD in conjunction with psychotherapy are still lacking. Currently, several ongoing clinical trials are aimed at evaluating the efficacy of cannabinoids as an adjunctive to PE therapy in PTSD patients, such as the study NCT03518801, which explores the effects of CBD, and the EudraCT 2020-001965-36, which explores the effects of a FAAH inhibitor. Although no evidence has yet been published regarding the effects of cannabis and synthetic cannabinoids in conjunction with psychotherapy on PTSD patients, there have been several studies aimed at investigating the effects of these compounds as a single treatment. Oral nabilone, a synthetic cannabinoid, prior to bedtime, was proven to reduce the frequency and intensity of nightmares in PTSD patients [176, 177]. Cessation of nabilone treatment re-established nightmares, which were again suppressed with re-dosing the drug, indicating that these effects can be specifically attributed to cannabinoid treatment [176]. Improvements in sleep time, reduction of daytime flashbacks, insomnia and elimination of night sweats were also observed in a retrospective study [178]. Δ^9^-THC administered orally at the dose of 5 mg twice a day was proven to decrease PTSD symptoms severity, including hyperarousal and frequency of nightmares, and to ameliorate sleep quality when administrated as add-on therapy [179]. The use of cannabinoids is associated with acute side effects, such as dry mouth, dizziness, fatigue, and nausea and vomiting at a higher dosage. Notably, the use of cannabinoids might be an additional risk factor for the development of psychotic disorders in susceptible individuals and cognitive deficits in adolescents [180, 181]. Therefore, more studies which might demonstrate the efficacy of these compounds on PTSD symptoms are needed to justify their possible use in clinical practice.

### 6.6 Psychotherapeutic Process Catalysts: Hallucinogens

#### 6.6.1 3,4-Methylenedioxymethamphetamine

##### 6.6.1.1 Animal Studies

3,4-methylenedioxymethamphetamine (MDMA) is a psychoactive substance that induces the release of serotonin and prevents the reuptake of NE, 5-HT, and DA by inhibiting the respective monoamine transporters and induces 5-HT efflux; it relies upon the disruption of vesicular monoamine storage by targeting the vesicular monoamine transporter 2 (VMAT2) [182-184] (Fig. **1**). MDMA also induces the hormonal release of oxytocin, cortisol, prolactin, and vasopressin [185, 186]. Structurally, MDMA is an amphetamine derivate with mood-elevating and prosocial proprieties. These enhancing effects on mood and social behavior have been identified as the main reason by which MDMA seems to improve the effects of psychotherapy on PTSD symptoms [187]. However, considering that other mood elevators, such as anxiolytics, did not report these ameliorative effects on PTSD symptoms it has been hypothesized that MDMA directly interferes with processes of extinction learning [188]. The effects of MDMA on fear extinction were first investigated in mice in a Pavlovian-cued fear conditioning and extinction paradigm [189]. MDMA (7.8 mg/kg; i.p.) given 30 min before extinction training reduced freezing across extinction sessions in mice previously exposed to tone-footshock pairings. In addition, mice previously dosed with either 5.6 mg/kg or 7.8 mg/kg of MDMA before extinction training and tested in the extinction context the following day showed enhanced extinction learning retention when compared to control and 3 mg/kg MDMA-treated mice. Extinction enhancement induced by MDMA persisted for at least 10 days and even when extinction retention was tested in an unfamiliar context. In other words, no renewal of fear memory was observed. Finally, MDMA administered immediately after extinction training did not cause any change in conditioned freezing at any dose tested [189]. A deeper understanding of the mechanisms by which MDMA enhances the extinction of fear memory is necessary to translate these important findings into humans. C-fos is an indirect marker of cellular activity whose mRNA upregulation indicates recent activity [190]. Long-lasting MDMA-induced reductions in conditioned freezing were related to changes in expression in the early-response gene c-fos within the amygdala and medial prefrontal cortex (mPFC) [189]. Reduced levels of brain-derived neurotrophic factor (BDNF) were correlated to high risk of developing PTSD and less responsiveness to exposure-based therapy in humans [191, 192]. In rodents, extinction learning was enhanced by increasing BDNF signaling in regions critically involved in fear extinction learning and retention, such as amygdala or mPFC [193]. It was found that MDMA increased BDNF expression in amygdala, but not in mPFC, only when combined with extinction training, whereas no effect on amygdala BDNF levels was found when MDMA was administered without being followed by extinction sessions [189]. Enhanced BDNF levels in the amygdala were proven to mediate the improving effect of MDMA on extinction training. A direct infusion of a BDNF neutralizing antibody in the amygdala before extinction training abolished the MDMA-mediated facilitation of extinction retention the following day [189]. Furthermore, a different study reported the involvement of the serotonin transporter (5-HTT) and the serotonin type 2A receptors (5-HT_2A_ R) in the mediation of MDMA's enhancing effect on fear memory extinction [194]. Mice dosed with MDMA prior to extinction training were pre-treated with citalopram (selective serotonin reuptake inhibitor, SSRI), reboxetine (norepinephrine reuptake inhibitor, NRI), or RTI-336 (dopamine transporter, DAT inhibitor). At the extinction retention test, the following day, only citalopram (SSRI), but not reboxetine (NRI), or RTI-336 (DAT inhibitor), prevented the MDMA-enhancing effect on extinction retention, revealing that MDMA-induced enhancements of extinction can be impaired by pharmacologically inhibiting the 5-HTT [194]. However, the only increase in extracellular levels of 5-HT in the brain is unlikely to account for MDMA's effect on fear memory extinction. Indeed, administration of fenfluramine – a compound that selectively enhances 5-HT release reversing 5-HTT function– 30 min prior to extinction training dose-dependently increased conditioned freezing during extinction training but did not affect extinction retention the following day [194]. In addition, citalopram treatment itself selectively increased extracellular 5-HT in the brain [195], but it did not enhance fear memory extinction [196, 197]. Moreover, the selective 5-HT_2A_R antagonist M100 administered 30 min prior to MDMA treatment prevented MDMA's positive effect during extinction training and the enhancement of fear memory extinction retention 24 h later. Thus, suggesting the importance of 5-HT_2A_R in mediating the MDMA's effect on extinction memory [194]. Fear-potentiated startle is a physiological reflex conserved across species [198]. Since fear-potentiated startle has also been used in humans, it may have a higher translational value compared to fear conditioning [199]. Mice, upon being exposed to tone-footshock pairings, underwent an extinction training phase and an extinction memory retention test the following day, during which startle stimuli alone and tone-startle stimuli pairings were delivered. They found that MDMA administered prior to extinction training dose-dependently reduced fear-potentiated startle during extinction sessions and only 7.8 mg/kg of MDMA prevented the expression of fear-potentiated startle during the extinction retention test the following day. Consistently to the above described study by Young *et al.* [194], the facilitating effect of MDMA in reducing fear-potentiated startle was abolished in mice chronically treated with citalopram for 23 days before the extinction training, which show downregulation of 5-HT_2A_R expression [200, 201].

##### 6.6.1.2 Human Studies

It is well-known from clinical practice that MDMA improves mood and euphoria, gives a sense of well-being, increases extroversion, openness to new experiences, sociability, self-confidence, and attachment behavior [202]. Therefore, patients can be highly involved during therapy sessions by increasing both their trust in clinicians and the patients’ willingness to face the traumatic memories. In particular, patients under the influence of MDMA seemed to recall the traumatic memories in a neutral and safe manner, without neither feeling distress or discomfort or inhibiting access to emotions [188]. In other words, MDMA maintains an optimal arousal state in which PTSD patients may stay emotionally engaged without being overwhelmed by anxiety while revisiting traumatic experiences [203]. Thus, traumatic memories are more prone to be updated and reconsolidated by mismatching the fearful and anxious retrieved memory to the positive affective state triggered by MDMA [204, 205]. The effect of MDMA has been extensively evaluated in a therapeutic method described in ‘*A Manual for MDMA-assisted Psychotherapy in the Treatment of PTSD*’ [206]. In a double-blind clinical trial, the efficacy of MDMA treatment (dosed at 125 mg plus an optional supplemental 62.5 mg) coupled with psychotherapy sessions was evaluated in PTSD patients [207]. To assess PTSD symptoms severity, the CAPS-4 was measured at baseline, and at different time points during the trial. The MDMA-treated group showed significantly lower CAPS-4 scores than the placebo group after only one MDMA session, which remained significantly lower 2 months after the second MDMA session [207]. To test whether the positive outcomes obtained in this study were sustained over time, 16 out of 19 patients, who were treated with MDMA, completed a long-term follow-up (17-74 months) [208]. Although 2 of the 16 patients had a relapse of PTSD symptoms, overall, there were no statistical differences between the mean CAPS-4 score at long-term follow up and the mean CAPS-4 score previously obtained at 2-month follow up. Thus, demonstrating that improvements in PTSD symptoms due to MDMA combined with psychotherapy treatment were sustained over time for most participants [208]. In a similar randomized, double-blind, active-placebo controlled trial, 12 subjects were enrolled and were dosed with low-dose (25 mg, plus 12.5 mg supplemental dose) or full-dose of MDMA (125 mg, plus 62.5 mg supplemental dose). Although no statistically significant reductions in CAPS-4 scores were found in full- compared to low-dose groups at 2-month follow-up, CAPS score improved at the 1-year follow-up for the full-dose group only [209]. Two more recent studies extended the aforementioned findings and supported the long-term symptoms’ improvement when combining MDMA with psychotherapy for treating PTSD, paving the way to phase III clinical trials [206, 210]. In one study, a randomized, double-blind, dose-response, phase 2 trial, 26 patients whose PTSD symptoms had lasted for 6 months or more and CAPS-4 score was 50 or greater at baseline, were dosed with 30 mg (active control), 75 mg or 125 mg of MDMA, and given psychotherapy sessions [206]. All participants were assessed 12 months after the last MDMA session [206], and they did not meet CAPS-4 PTSD criteria any longer. The 75 mg and 125 mg groups had significantly greater improvements in PTSD symptom severity (CAPS-4) than the 30 mg group. Secondary outcomes assessment at 1 month follow up reported that depression symptoms for the 125 mg group, but not for 75 mg group, were significantly reduced compared with the 30 mg group. All scores of secondary measures showed improvement compared with baseline at a 12-month follow-up [206]. The same findings of results in the primary outcome (CAPS score) at 1-month follow up, were found in another double-blind study in which 28 patients were randomized to receive two active doses (100 mg or 125 mg) or a low dose (40 mg) of MDMA administered during eight-hour psychotherapy sessions [210]. Although MDMA has been associated with neurotoxicity and abuse liability, none of these concerns were detected during medical supervision in clinical use [188, 206, 207]. Patients treated with the first dose of MDMA frequently reported anxious episodes and depressed moods that were easily dealt with psychological support. More frequently, headache, fatigue, hyperthermia, hypertension, and increased heart rate were reported. Therefore, the use of MDMA in patients with cardiovascular disease could not be recommended.

#### 6.6.2 Ketamine

##### 6.6.2.1 Animal Studies

Ketamine, a phencyclidine derivative, is one of the most commonly used drugs in anesthesia [211]. Racemic ketamine is a mixture of (S)- and (R)-ketamine, but (S)-ketamine carries roughly 3- to 4-fold greater potency as an anesthetic but also most of the psychotogenic side effects [212]. As a nonselective NMDA receptor antagonist (Fig. **1**), ketamine seems to be beneficial in some psychiatric disorders, while for others, such as PTSD, its efficacy is still far from being accepted [213, 214]. It has been demonstrated in rats that ketamine anesthesia immediately after a stressful event increases memory consolidation of the trauma [215], and that this effect is mediated by a combined peripheral-central sympathomimetic action [216]. However, only a few preclinical studies have investigated the effects of ketamine on fear memory extinction and reconsolidation. Ketamine (10 mg/kg; i.p.), injected before extinction sessions, facilitated contextual fear extinction in adolescent mice [217]. In a separate study, chronic injections of ketamine (22 days) in combination with extinction training suppressed the anxiety-like behavior and fear relapse in traumatized mice. Further, ketamine effects on fear extinction seemed to be mediated by increased BDNF levels in the mPFC and hippocampus of traumatized mice through down-regulation of the methylation of BDNF exon IV [218]. In addition, ketamine, in conjunction with extinction exposure, increased levels of mammalian target of rapamycin complex 1 (mTORC1) and c-fos in the mPFC, and infusion of the selective mTORC1 inhibitor rapamycin into the mPFC blocked the effects of ketamine on fear extinction [219].

##### 6.6.2.2 Human Studies

The beneficial effects of ketamine on PTSD symptoms were first evaluated by Feder and colleagues [220]. A single intravenous (i.v.) infusion of 0.5 mg/kg of ketamine together with an i.v. infusion of 0.045 mg/kg of midazolam in 41 patients with chronic PTSD and associated depressive symptoms led to a significant but transient reduction of PTSD symptoms. Therefore, the idea of obtaining a long-lasting remission with ketamine came up to treat PTSD patients, as well. By using a mindfulness-based cognitive therapy, patients with refractory PTSD were asked to recall their fear memories before the infusion of a single i.v. dose of 0.5 mg/kg ketamine (or saline) over 40 min. In addition, in the peri-infusion period, 2 cycles (10 min each) of combined extinction-reconsolidation interventions were practiced by the subjects for extinguishing traumatic memories and reconsolidating calming/healthy memories [221]. Ketamine induced a more durable reduction in PTSD symptoms, as shown by the CAPS-4 score than placebo (34 days ketamine *vs*. 16 days placebo). It was estimated that this time period is 5-times longer than those reported initially by Feder and colleagues [220], in which only a single administration of ketamine was dosed. Noteworthy, as a dissociative anesthetic agent, ketamine causes a detachment from reality with a relaxed and dissociated mental state in which subjects do not react to the traumatic memories and, as happens with MDMA, patients have the possibility to maintain a detached and engaged emotional state without exceeding in anxiety while revisiting the traumatic experience. However, studies reporting improvements in PTSD symptoms induced by ketamine-psychotherapy association are still poor and the usage of ketamine in this clinical practice seems to be limited by its short-lived beneficial effects. In addition, considering the reported side effect of ketamine, such as increased heart rate and blood pressure, anxiety episodes, nausea, visual and perceptual alterations, the use of this agent is not yet recommended [222].

#### 6.6.3 Other Psychedelics

Psylocibin, lysergic acid diethylamide (LSD) and dimethyltryptamine share a common mechanism of agonism at serotonergic 5-HT2A receptor. Although few studies have indicated possible use of these psychedelics for depression [223, 224] and obsessive-compulsive disorder [225], no other study has yet been reported regarding a potential treatment for PTSD. However, the effects on empathy [226], mindfulness related capacities [227], avoidance, acceptance and connectedness [228], long-term openness [229, 230], and emotional break-through experiences [231] along with preclinical studies, demonstrating an enhancing effect on fear memory extinction [232, 233], should determine a renewed interest on these compounds.

## CONCLUSION

The present review summarizes encouraging data from both animal and human studies supporting the efficacy of exposure-based therapy combined with pharmacological treatments in PTSD. The compounds herein presented are characterized by different and complex mechanisms of action, which, in some cases, have not yet been fully clarified. The efficacy of PE therapy relies on repeatedly exposing patients to feared stimuli engaging in extinction or reconsolidation of traumatic memories. Drugs may act on these two memory processes either through enhancing extinction learning or by blocking fear memory reconsolidation. The extinction of a consolidated memory consists in acquiring new safe information that can block and correct maladaptive behavior when patients face their fear provoking stimuli. Therefore, extinction mechanisms are not much different from those of consolidation of newly acquired information. Drugs that improve the consolidation of new information frequently enhance the extinction process as well. The enhancement of extinction learning represents one of the most promising approaches for treating PTSD, although some caveats might emerge. High fear state by the end of an exposure session could lead to the reconsolidation of fear memory in a stronger state, worsening, instead of improving, PTSD symptoms as described for DCS, DEX or MB. Furthermore, to exert appropriate effects, drugs must be administered in the proximity of (before or immediately after) the exposure sessions in a timely manner, taking into consideration the time frame of extinction memory mechanisms and the pharmacokinetic properties. It should be taken into account that the majority of the drugs interfere with memory processes, often in a dose-dependent linear or U-shaped curve, thus the usage of appropriate doses is of the utmost importance to avoid inefficacy or opposite effects with respect to those expected. Preferably, the administration of a drug facilitating the extinction of trauma should be done when patients’ emotional state is not heightened by the psychological treatment itself. This can be verified after the extinction session when the patient’s response can be precisely determined. For instance, MB, having efficacy with post-session administrations, offers the possibility to be dosed in this way; howewer, the majority of the other drugs do not. Alternatively, to prevent the reconsolidation of aversive memories due to patients’ excessive fearful state at the time of drug administration, it would be wise to postpone the administration of the extinction enhancer after the first/second exposure session when patient’s response has been precisely characterized and stabilized. Besides the drugs directly acting on extinction or reconsolidation processes, there are others known as psychotherapeutic process catalysts that have shown promising results, such as MDMA. The improving impact on PTSD psychotherapy of these latter substances, seems to be due to a mood elevator effect that maintains an optimal arousal state in which PTSD patients may stay emotionally engaged without being overwhelmed by anxiety while revisiting traumatic experiences. In addition, psychotherapeutic process catalysts increase the trust in clinicians and the patient’s openness leading to a strong recall of traumatic memories during the exposure sessions. These effects could be particularly beneficial in patients with PTSD aggravated by alexithymia, which is a condition characterized by the inability to recognize and talk about their emotions [234]. Besides all drugs described herein, many other non-pharmacological approaches such as deep brain stimulation, transcranial magnetic stimulation or physical exercise have been recently proposed for their potential to improve psychotherapy outcomes [235-240]. However, more studies are needed to provide additional convincing evidence for their safety, efficacy and possible beneficial effects of their potential combination with psychotherapy. Nowadays, there is an arisen interest in the scientific community in PTSD research, which is growing hugely because of the COVD-19 pandemic [241]. Indeed, the prevalence of PTSD after infectious disease pandemics was found to be even higher than the estimated prevalence after other disasters. Who faced enormous physical and mental pressures as healthcare workers or who experienced anxiety and distress associated with quarantine were found to have a higher potential of developing post-pandemic PTSD [242]. In this perspective this current review may pave the way for additional studies aimed at improving current therapies for treating this disorder.

## LIST OF ABBREVIATIONS

BBB: Blood-Brain Barrier
BDNF: Brain-Derived Neurotrophic Factor
BLA: Basolateral Amygdala
cAMP: Cyclic Adenosine Monophosphate
CB1: Cannabinoid Type 1
CBT: Cognitive Behavioral Therapy
GCs: Glucocorticoids
GR: Glucocorticoid Receptors
LSD: Lysergic Acid Diethylamide
PE: Prolonged Exposure
PTSD: Post-traumatic Stress Disorder
VMAT2: Vesicular Monoamine Transporter 2
